# Spatio-temporal variations of the major meteorological disasters and its response to climate change in Henan Province during the past two millennia

**DOI:** 10.7717/peerj.12365

**Published:** 2021-11-02

**Authors:** Xiang Li, Hui Lu, Zhaokang Zhang, Wei Xing

**Affiliations:** 1 College of Geographical Sciences, Fujian Normal University, Fuzhou, Fujian, China; 2 School of Economics and Management, Sanming University, Sanming, Fujian, China; 3 National Park Research Center, Sanming University, Sanming, Fujian, China; 4 College of Geography Science, Qinghai Normal University, Xining, Qinghai, China; 5 College of Geographic Sciences, Xinyang Normal University, Xinyang, Henan, China

**Keywords:** Historical documents, Meteorological disaster, Spatio-temporal variations, Climate change, Henan Province

## Abstract

In China, historical documents have recorded large quantities of information related to natural disasters, and these disasters have had long-lasting effects on economic and social activities. Understanding the occurrence of the natural disasters and their spatio-temporal variation characters is crucial for sustainable of our society. Therefore, based on the collection and collation of historical documents, and adopting mathematical statistics, Kriging interpolation, correlation analysis and other methods, we systematically explored the meteorological disasters in Henan Province during the past two millennia in analyzing their spatio-temporal distribution characters and driving forces. The results demonstrate that there were five major types of meteorological disasters in Henan Province, including drought, flood, hails, low temperature and frost and insect pests, which presented obvious spatio-temporal variations and have occurred frequently during the past two millennia. According to the historical documents, the major meteorological disasters occurred 1,929 times in Henan from 221 BCE to 2000 CE. On the whole, the disaster frequency show that the occurrence cycle of the meteorological disasters has obvious changes, which mainly occurred in the middle and late stages during the past two millennia, especially after 1300 CE. Furthermore, we also find that the variation of meteorological disaster events is consistent with the variation of temperature in eastern China and the frequency of meteorological disaster increases in the cold period, but decreases in the warm period. In addition, there are obvious differences in the spatial distribution of the major meteorological disaster, which were mainly distributed in the northwest and southern part region of the Henan Province before 1911 CE. While after 1911 CE, the northern and southeastern parts were the meteorological disaster-prone areas in this region during this period. Spatial correlation analysis of each meteorological disaster before and after 1911 CE points out the droughts disaster frequency-occurring district has transferred in different periods, while the hail and low temperature and frost disasters just have a smaller transferred during these two periods. Conversely, the frequency-occurring districts of floods and insect pest disasters have no obviously transferred in different periods. These results can provide an important scientific basis for governmental decision makers and local people to prevent and mitigate meteorological disaster in the future.

## Introduction

Meteorological disasters are one of the most serious issues and costly natural disasters recognized in the world (Intergovernmental Panel on Climate Change (IPCC), 2014; Shi, Cui & Tian, 2020; Wu et al., 2018b). The existence of a close and complicated correlation is evident between meteorological disaster and economic fluctuation, social harmony and crisis, and even dynastic transitions in historical periods (Lee et al., 2017; Pederson et al., 2014; Lee & Zhang, 2013; Buckley et al., 2014; Lee & Zhang, 2010). Therefore, the study of historical meteorological disasters is one of the important contents of the “Past Global Changes (PAGES)” (Turney et al., 2019) and “Climate Variability and Predictability Programmers (CLIVAR)” (Zhou & Wu, 2017). Before the instrumental era, historical documents have been used to describe the meteorological disasters of high temporal resolution (*i.e.*, drought, flood) around the world, which focused on the frequency and magnitude of the meteorological disasters and analyze their impact on society (Shi, Cui & Tian, 2020; Wu et al., 2018b; Lee et al., 2017; Pederson et al., 2014). It is well known that the meteorological disasters have occurred running through mankind’s history (Wu et al., 2018b; Lee et al., 2017; Zheng, Wen & Fang, 2020; Fang et al., 2015). Compared with ancient times, with the progress and improvement of scientific and technological, modern humans’ meteorological disaster monitoring and early-warning abilities have been significantly improved. However, the study of meteorological disasters in ancient times can reflect the variation history of meteorological disasters more comprehensively, and has been an important scientific reference for analyzing and predicting the formation and variation characteristics of meteorological disasters more accurately for the future (Liu & Yang, 2021; Ge et al., 2012). Furthermore, it is also of great practical significance to explore the factors affecting the change of the meteorological disasters in the historical period for understanding the long-term change rule and trend of disasters, as well as for current meteorological disaster prevention and mitigation (Lee et al., 2017; Zheng, Wen & Fang, 2020; Wu et al., 2018b).

Henan Province is located in the plain area of the middle and lower reaches of the Yellow River, and is located in the transitional zone of northern subtropical and warm temperate humid monsoon in eastern China. Due to its unique geographical location, it makes meteorological disasters become one of the natural disasters that have the greatest impact on agricultural production and people’s lives and property in this region (Li et al., 2017a). Furthermore, Henan is one key birthplace of ancient Chinese culture, and is known as “the cradle of Chinese civilization” (Shu & Finlayson, 1993; Li, Li & Zhang, 2020). Therefore, Henan Province has a long history with abundant written documents about natural disasters and anthropogenic events, such as droughts, flood and epidemics. Ancient people learnt to record disasters in the Shang Dynasty in this region, and then conducted more systematic records in the Spring and Autumn period (Liu, Yang & Li, 2012). Such documents are generally continuous and reliable in the official historical books, which are thus the source available of quantitative meteorological, economic fluctuation; cultural, and social harmony and crisis information (Ge et al., 2016; Ge, Zheng & Hao, 2015), which provide important evidence for discussing the features and changing characteristics of meteorological disasters in the historical period.

In the recent years, research regarding historical disasters have been carried out and obtained remarkable results through the historical documents. This verifies the reliability of the historical documents in assessing the historical disaster records. For instance, on the basis of the historical documents, Wu et al. (2018b) analyzed and compared the spatial-temporal differentiation of disasters and correlation between various major natural disasters during the Ming & Qing Dynasty in the Chaohu Lake Basin. Similarly, Li, Li & Zhang (2020) presented a decadal resolved Yellow River basin flooding frequency record during the past two millennia, and synthesized historical and climatic records in the Yellow River basin to investigate their relationship. Zhang et al. (2018b) analyzed the spatio-temporal variation characteristics of drought disasters in Guanzhong region and its response to the Little Ice Age, where good correspondence between the drought disaster and the secondary fluctuations of cold and warm in the Little Ice Age in the Ming & Qing Dynasty was established. Furthermore, on the basis of historical literature data, some scholars have also analyzed the impact of extreme meteorological disasters on agriculture, livelihoods, and socioeconomic systems (Zheng et al., 2014b; Xiao & Yan, 2019). It can be seen from the above analysis that the spatial-temporal characteristics of meteorological disasters is a core content in Chinese historical natural disaster research, and some scholars have done lots of remarkable work on theoretical and empirical aspects of natural disaster. Through previous studies mainly focused on statistical analysis of single disasters (Wu et al., 2018b; Lee et al., 2017; Wu et al., 2018b; Zhang et al., 2018b; Wei et al., 2016) on specific areas (Wu et al., 2018b; Li, Li & Zhang, 2020), or a particular period such as Little Ice Age (LIA) (Wu et al., 2018b; Liu & Yang, 2021; Zhang et al., 2018b; Xiao & Yan, 2019). That there are still very few comprehensive studies reported on meteorological disasters in the provincial or larger scales of China.

Henan Province is one key birthplaces of ancient Chinese culture. Historical documents about natural disasters in this region were quite completely recorded in terms of occurrence time, location and various types of disasters (Li et al., 2017b; Li, Li & Zhang, 2020; Liu, Yang & Li, 2012). Therefore, relevant studies on the spatio-temporal variation characteristics of the meteorological disasters in Henan have been documented. For instance, through arrangement, classification and statistical analysis of historical data on drought disasters during the Ming and Qing Dynasties, Zhang et al. (2018c) and Li et al. (2015a) explored the spatio-temporal distribution characteristics of drought events from both the frequency and intensity. Moreover, by classifying and sorting the flood disaster records of Henan area from CE 1616 to 1911, the spatio-temporal difference rules of the abnormal flood and the response of the occurring flood to the East Asian summer monsoon and the variation of the solar activity was discussed (Li et al., 2017a). Additionally, based on the observed meteorological data, the spatio-temporal evolution, intensity, and the primary causes of drought occurrence in Henan Province were revealed (Shi et al., 2015); studies found that the SPEI (standardized precipitation evapotranspiration index) values effectively reflected the spatial and temporal pattern of drought occurrence. By comparison, it can be found that the previous studies on the meteorological disasters mainly focused on the recent decades since the 1950s in Henan Province, and largely based on modern instrumental data. Furthermore, the study on historical meteorological disasters mostly focused on statistical analysis of single disasters in Henan Province, such as droughts and floods (Li et al., 2017a; Li et al., 2015a), while some scholars reported the spatial and temporal characteristics in a particular period (Shi et al., 2015; Li et al., 2015a; Xing et al., 2020; Zhang et al., 2021b) or a specific region (Chu & Zhao, 2015; Li et al., 2017b). However, there are few reports on the spatio-temporal variation characteristics of meteorological disasters and their driving force on a long time scale in Henan area.

Therefore, in this study, we systematically collected and collated the abundant natural disasters based on the historical documents. Then, by adopting mathematical statistics, ArcGIS spatial analysis and other methods, we present a 2,221-year meteorological disasters frequency record of the Henan Province from 221 BCE (the beginning of Imperial China) to 2000 CE. Meanwhile, by synthesizing climatic records, we aim to understand spatio-temporal characters of major meteorological disasters and driving forces in this region during the past two millennia. In addition, we focus on the changes of meteorological disasters over the past 2,000 years, as is because this period includes not only the period of fluctuation dominated by the rate of natural variability before the industrial revolution, but also the period of human influence superimposed on the basis of natural variability since the industrial revolution (Li, Li & Zhang, 2020; Ge, Fang & Zheng, 2014). Therefore, the research on the changes of meteorological disasters during the past two millennia can not only reveal the spatio-temporal distribution characteristics in this area, but also lay a foundation for analyzing whether human activities can have an impact on the occurrence of meteorological disasters, which has an important reference for grasping the occurrence rules of natural disasters.

## Materials and Methods

### Study area

**Figure 1:**
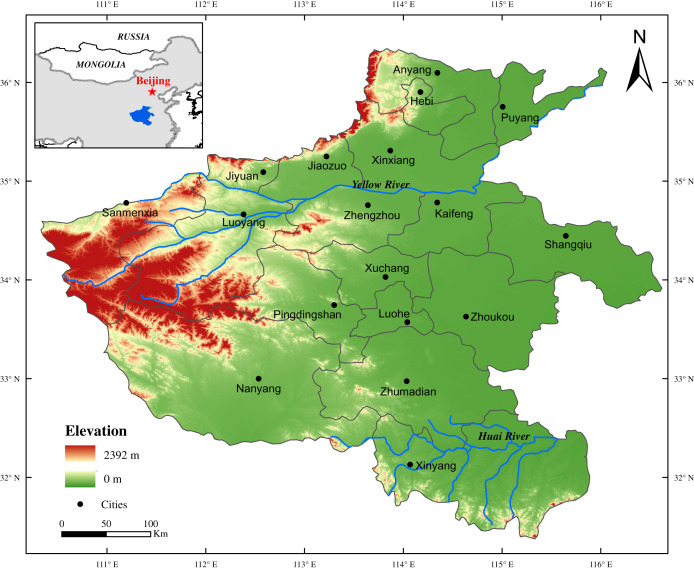
Study area and location. The geographic location of the Henan Province in China.

Henan Province is located in the central and eastern China, with an area of 16.7 × 10^4^ km^2^. The Yellow River and Huai River passes through this region, and the topographically, it lies in the transitional zone from the second stair to the third stair of China (Fig. 1). Additionally, the area is climatologically situated in the transition between northern and southern China, which is reported to be highly sensitive to climate changes (Zhang et al., 2018a; Miao, Ding & Wang, 2009). The transitional zone of northern subtropical and warm temperate humid monsoon climate in this area is characterized by a mild climate, significant monsoon with a well-defined rainy season, good heat condition, and long frost-free season. The climate is cold in winter (−1.8 to 1.6 °C, January) and hot in summer (26.9–27.8 °C, July) in this region, and the annual precipitation increases from 450 mm in the northwest to 600 mm in the southeast in this region, with great differences in regional distribution and inter-annual variation. Because the area has the characteristics of east-west transition and north-south transition in terms of climate, geomorphology, vegetation and other geographical elements, it is vulnerable to ecological environment, sensitive to climate change, and prone to natural disasters (Gao, Li & Cui, 2017).

### Data sources and processing methods

#### Historical meteorological disasters records

Documentary records have unique advantages for analyzing past meteorological disasters, which are characterized by high spatial and temporal resolution, precise dating and clear climatic significance (Ge, Zheng & Hao, 2015; Zheng et al., 2014a; Zheng et al., 2021). Recently, many scholars have done a lot of research on meteorological disasters by using historical documents.

In this study, the meteorological disaster record in Henan area are from official historical archives that were summarized in two books, that is, “*Compendium of Chinese Meteorological Records of the Last 3,000 Years*” (Zhang, 2000) and “*Encyclopedia of China’s Meteorological Disasters: Henan Volume*” (Wen, 2005). The first book was authored by Deer Zhang in 2000, and it contains records of local weather or climate conditions and related disaster records from the 13th century BCE to 1911 CE. Another, by Kegang Wen in 2005 which records droughts, floods, low temperatures and frost, typhoons and other meteorological disasters taking years as the unit. While collating the data, we made statistics on the disasters with clear records of the time, region and disaster situation in the historical documents, to form the database. Furthermore, we also used the local chorographic that contain the Henan area disasters records to verify and supplement our dataset, which can reduce the likelihood of the meteorological disasters records not being fully collected. In addition, administrative divisions have often changed in Henan area during the past times. Considering the long-time span of the study, we referred the map of Henan Province in 2018 as the base map in this study (Fig. 1), in an effort to reduce the errors caused by regional changes in data statistics. This treatment can also revise the location of meteorological disasters in each historical record corresponding to the modern political map. For example, Mianchi County in Henan Province during the Ming Dynasty was found to be Sanmenxia City in today’s Henan Province, and we believe that this disaster occurred in Sanmenxia City. In addition, we also used the reconstruction of temperature and precipitation series from the historical period of the relevant region (Ge et al., 2012; Zheng et al., 2014a).

#### Analytical methods

In this study, the geological succession method is used for the statistical analysis, that is, for an area with exact records regarding the number of disaster occurrences, we calculate m (times) of m (place) with the year based on different areas; for a disaster which happened in different areas during the same period, it will be recorded according to the number of areas, *i.e*., m (times) for m (place) number of disasters in different cities is measured by time records. The above method was used in the statistics of the number of all the disasters.

Furthermore, as noted in the historical documents, merely discontinuous meteorological disasters events were recorded during the Zhou Dynasty, while the historical documents were more detailed and easier to compare after 221 BCE (the beginning of Imperial China), and since then meteorological disasters records were documented officially and accurately. Meanwhile, all meteorological disasters records in the official documents are evidenced with crucial information such as precise dates, accurate places and exact impacts. For example, as noted in “*Earlier Song* *Dynasty: the Five dynasty Huiyao*”, a drought that occurred in the spring and summer of 943 CE in Kaifeng Prefecture, Dongjing (today’s Kaifeng city) recorded countless starving people and wanderers. Moreover, since the modern times, with the introduction of newspapers and other media, the statistical data of disasters are relatively complete. According to this scenario, our paper divides the history into two periods taking the year of 1911 CE (when the Republic of China was founded) as the breaking point to make a better comparative analysis. Then, by using the statistical analysis, Kriging interpolation and other methods, the spatio-temporal characters of the major meteorological disasters and driving forces in Henan area during the past two millennia are discussed.

## Results

### Occurrence frequency of the meteorological disasters

We have compiled a 2,221-year meteorological disasters history of the Henan Province from 221 BCE to 2000 CE. Meanwhile, based on the direct economic losses and occurrence times, these five major meteorological disasters were selected as the research objects, including drought, flood, hail, low temperature and frost and insect pests. On the whole, the major meteorological disasters occurred 1,929 times in Henan from 221 BCE to 2000 CE, and the frequency varies for different disasters, which accounted for 31.00%, 25.72%, 11.77%, 10.26% and 11.25%, respectively (Table 1).

**Table 1:** The number of the major meteorological disaster frequency in Henan Province during the past two millennia.

The type of disaster	Frequency (times)	Proportion (%)
Drought	598	31.00
Flood	689	35.72
Hail	227	11.77
Low temperature and frost	198	10.26
Insect pests	217	11.25

### The inter-decadal variation characteristics of the major meteorological disasters occurrences

**Figure 2:**
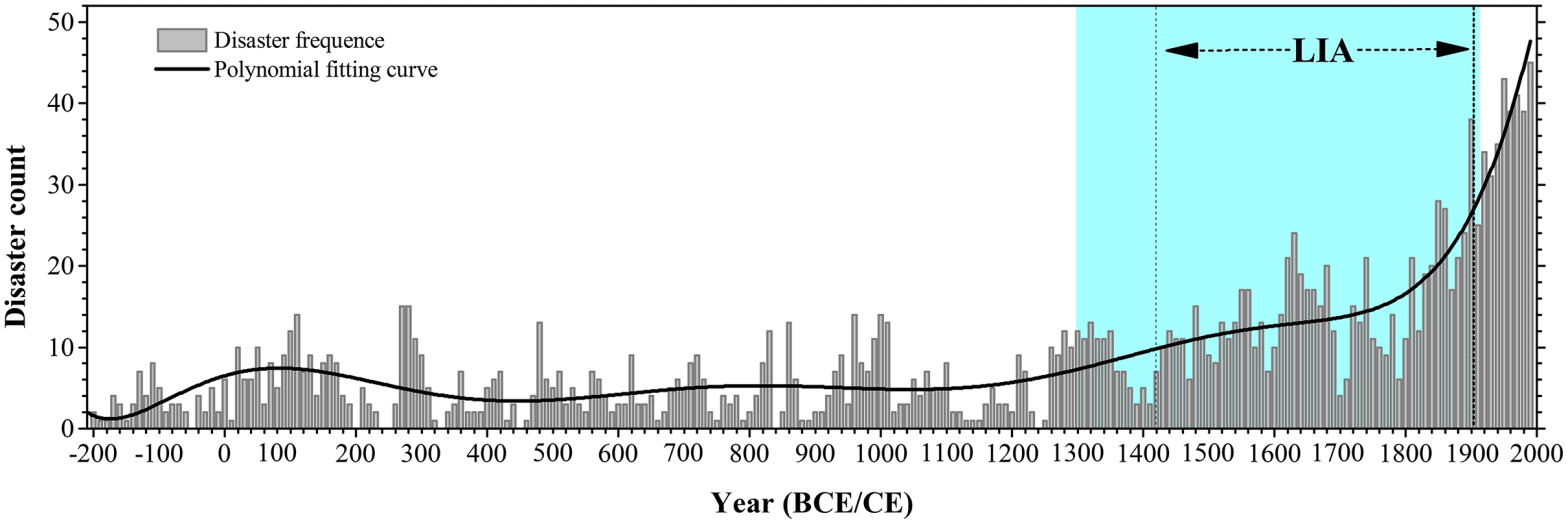
Major meteorological disasters frequency records. Major meteorological disasters frequency records and curve fitting in Henan Province from 221 BCE to 2000 CE (a 20-year resolution) in the historical documents. Vertical shading (light pink) denotes the period of high occurrence of meteorological disaster, Litter Ice Age (LIA) period was shown between the two vertical dash lines (1420–1910 CE) (Ge et al., 2012; Zheng et al., 2014a).

We calculated the decadal variation of the major meteorological disasters in Henan Province at a 20-years resolution (units: number of disasters). The results showed that the occurrence cycle of the major meteorological disasters has obvious changes during the historical period in this region (Fig. 2). As shown in the Fig. 2, two distinct stages can be identified based on the major meteorological disasters frequency records during the past two millennia: an early stage of low-frequency major meteorological disasters from the 220s BCE to 1300s CE; and a late stage of high-frequency major meteorological disasters during the 1300s–1910s CE. Before 1300s CE, the frequency of the major meteorological disasters was relatively low, especially between 200 BCE and 0 and between 400s and 900s CE. Thereafter, the occurrence frequency of the major meteorological disasters showed an obvious upward trend after 1300s CE, especially since 1800s CE. Meanwhile, we found that the variation of the major meteorological disaster events was consistent with the variation of temperature and moisture records in eastern China, and the frequency of the major meteorological disasters increased in the cold period, but decreased in the warm period. The major meteorological disasters were more likely to occur during the Dark Age Cold periods, as well as during the coldest period of the LIA (Fig. 2). In addition, it is to be noted that the major meteorological disasters frequency increased greatly since the foundation of the Republic of China (1911 CE) due to the intensive human activities as well as climate changes (Fig. 2).

### Spatial distribution characteristics of major meteorological disasters occurrences

The frequency of the major meteorological disasters in Henan Province during the past two millennia was statistically analyzed and the spatial distribution map was drawn based on the urban area. The results showed that the spatial distribution of the major meteorological disasters were uneven in this region. That the major meteorological disaster occurred 1,598 times in total before 911 CE in Henan Province, drought and flood disaster were characterized by high frequency and wide distribution (Table 2, Fig. 5). The areas with frequent meteorological disasters are complex with diverse landforms, densely populated and economically developed areas in this region. Since 1911 CE, the major meteorological disaster occurred 331 times in total, with the high incidence rate at the northern and western part in this region, while the drought and flood disaster were still the most important and frequent meteorological disasters in this region (Table 2, Fig. 6). From the perspective of the whole Henan, droughts were concentrated in the north and northwest of Henan Province during the past two millennia, showing the distribution characteristics of more droughts in the north and less in the south Luoyang City, in the north, was the most frequent area of droughts in the whole. However, floods were concentrated in the southern parts of Henan Province, especially in the Huai River Basin. Additionally, other major meteorological disasters also present obvious difference in Henan area, and mainly distributed in the northern region.

**Table 2:** The number of occurrences of each meteorological disaster in Henan Province during the past two millennia.

Types of disaster	Before 1911 CE		Since 1911 CE	
	Times	Frequency	Times	Frequency
Drought	522	0.24	76	0.85
Flood	617	0.27	72	0.81
Hail	160	0.07	67	0.75
Low temperature and frost	127	0.06	71	0.80
Insect pests	172	0.08	45	0.51

## Discussion

### Driving forces of the major meteorological disasters during the past two millennia

**Figure 3:**
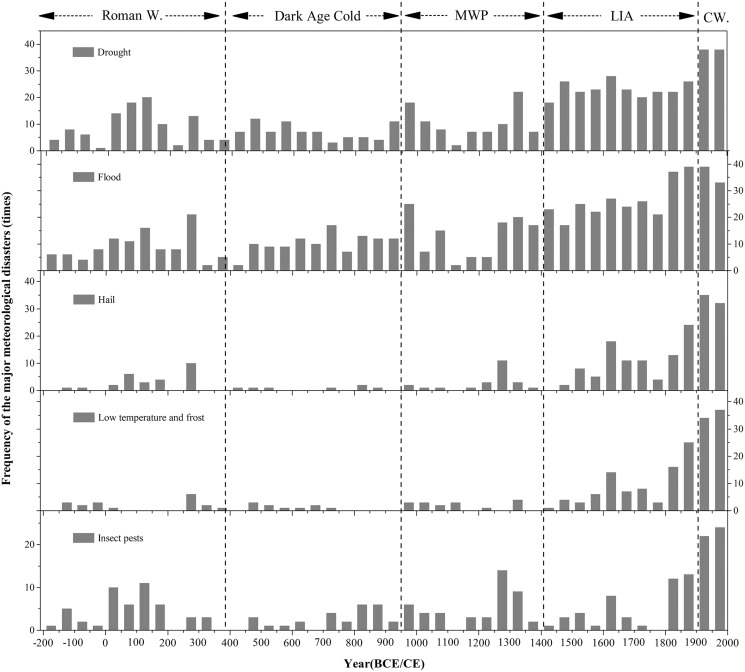
Major meteorological disasters for each 50-year in Henan Province. The frequency of the major meteorological disasters for each 50-year in Henan Province during the past 2,000 years. The dashed lines indicated the synthesis of the centennial warm/cold periods revealed from different publications since 2008 (Ge et al., 2012). Dash arrow: the approximate duration of Roman Warm, Dark Age Cold, Medieval Warm Period, Litter Ice Age (LIA), and Current Warm (CW) identified by (Lamb, 1997).

Based on the historical documents, we obtained the frequency of the meteorological disasters in Henan Province during the past two millennia. Statistically, it was found that the drought, flood, hail, low temperature and frost and insect pests were the main meteorological disasters in this region and occurred 1,929 times in total, among which drought and flood disaster occurred most frequently (Table 1, Fig. 3). Meanwhile, our analysis reveal that the major meteorological disasters became more and more frequent (Figs. 2 and 3), and the occurrence of the major meteorological disasters has an obvious stage changes in Henan during the past 2,000 years (Figs. 2 and 3). Comprehensive analysis revealed that this trend can be attributed some factors: (1) the special geographical position of the Henan Province; (2) the increasing population in areas dominated by agriculture; (3) the impact of climate change on centennial times scale.

It has been clearly indicated that the Henan Province was the main seriously-hit area of meteorological disasters in the history of China (Liu, Yang & Li, 2012). Our results revealed the drought and flood disasters occurred frequently in Henan during the past two millennia (Fig. 3), which was consistent with other results of historical major natural disasters research in the same and neighboring regions (Liu, Yang & Li, 2012; Zhang et al., 2018c; Wan et al., 2017c; Gao, Yin & Li, 2019; Hao et al., 2017). Statistics show that there were 689 times flood disaster in Henan Province during the past 2,000 years, accounting for 35.72% of the total, with an average of 15 times per 50-year, while drought disasters occurred a total of 598 times, accounting for 31.00%, with an average of 13 times per 50-year (Table 1). We hold an assumption that this phenomenon was related to the regional climate and landform in Henan Province. It is wildly known that the Henan area is located in the hinterland of the Central Plains of China, with the Yellow River and Huai River passing through this region (Fig. 1). As mentioned earlier, the Henan Province is climatologically situated in the transitional zone between northern and southern China, which shows the transition from warm temperate zone to north subtropical climate, and from the east plain climate to the west hilly and mountain climate (Shi et al., 2015). The transition is reported to be highly sensitive to climate changes (Shi et al., 2015; Miao, Ding & Wang, 2009; Zhang, Chen & Zhang, 2012). Affected by monsoon climate, the precipitation in this region shows the characteristics of great inter-annual variation and uneven distribution within the year (Shi et al., 2015; Gao, Yin & Li, 2019), which can easily cause the frequent occurrences of meteorological disaster, such as drought, flood, etc.

**Figure 4:**
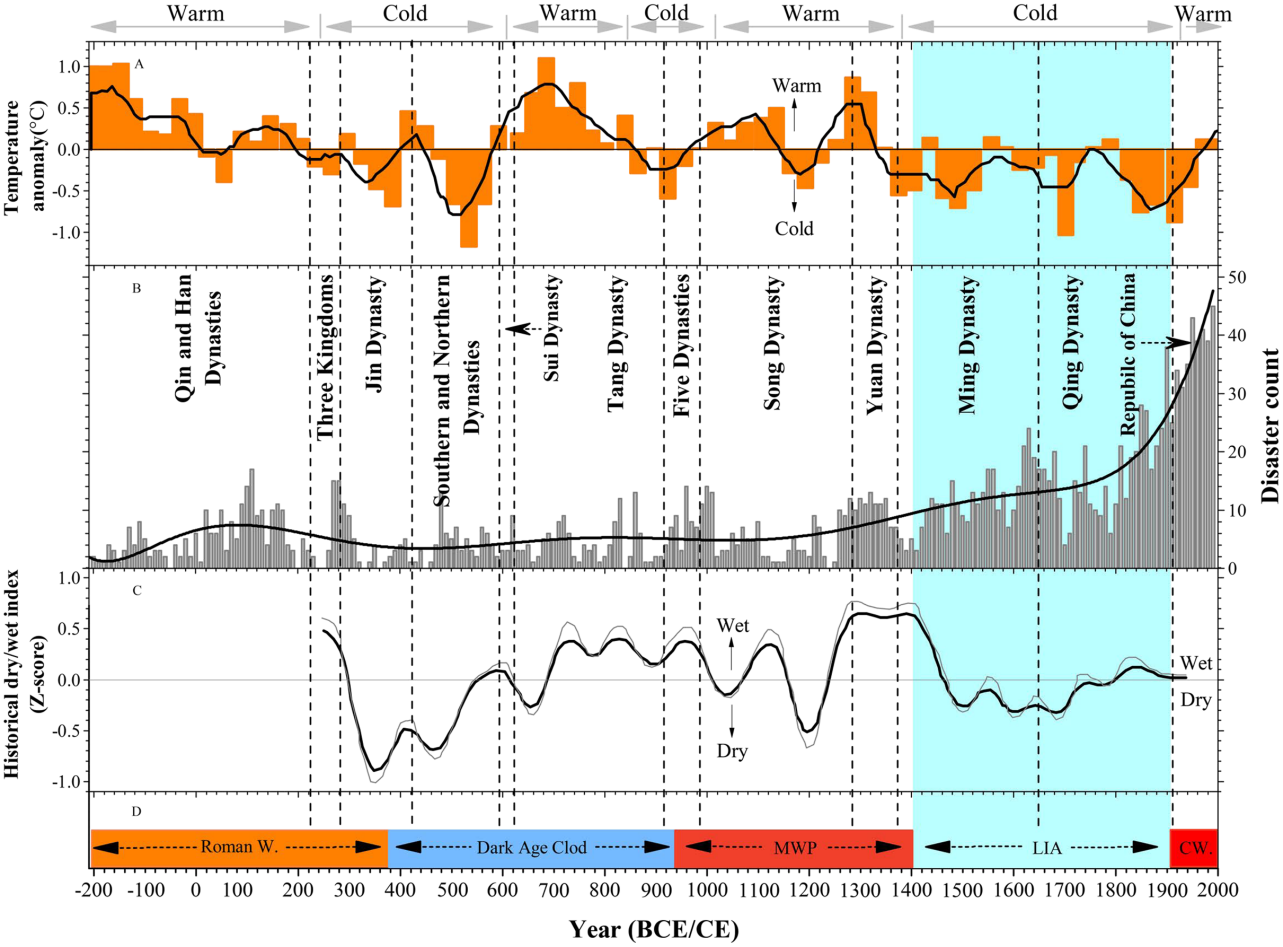
Sequences of temperature, disaster frequency and warm/cold periods from 210 BCE to 2000 CE. Sequences of temperature, disaster frequency and warm/cold periods from 210 BCE to 2000 CE. (A) The winter half-year temperature anomaly (30-year resolution) in eastern China during the past 2,000 years (bold line: 90-year running mean) (Ge et al., 2012). The gray arrows indicate a synthesis of the centennial warm/cold periods revealed by East China temperature series (Zheng et al., 2014a). (B) Major meteorological disasters frequency records and curve fitting in Henan Province from 221 BCE to 2000 CE. The dashed lines denote the start or the end of the Chinese dynasties (Zheng et al., 2014a). (C) Dry/wet index in the middle and lower Yellow River basin based on historical documents (Gong & Hameed, 1991). (D) Dash arrow: The approximate duration of Roman Warm, Dark Age Cold, Medieval Warm Period, Litter Ice Age, and Current Warm identified by (Lamb, 1997).

**Figure 5:**
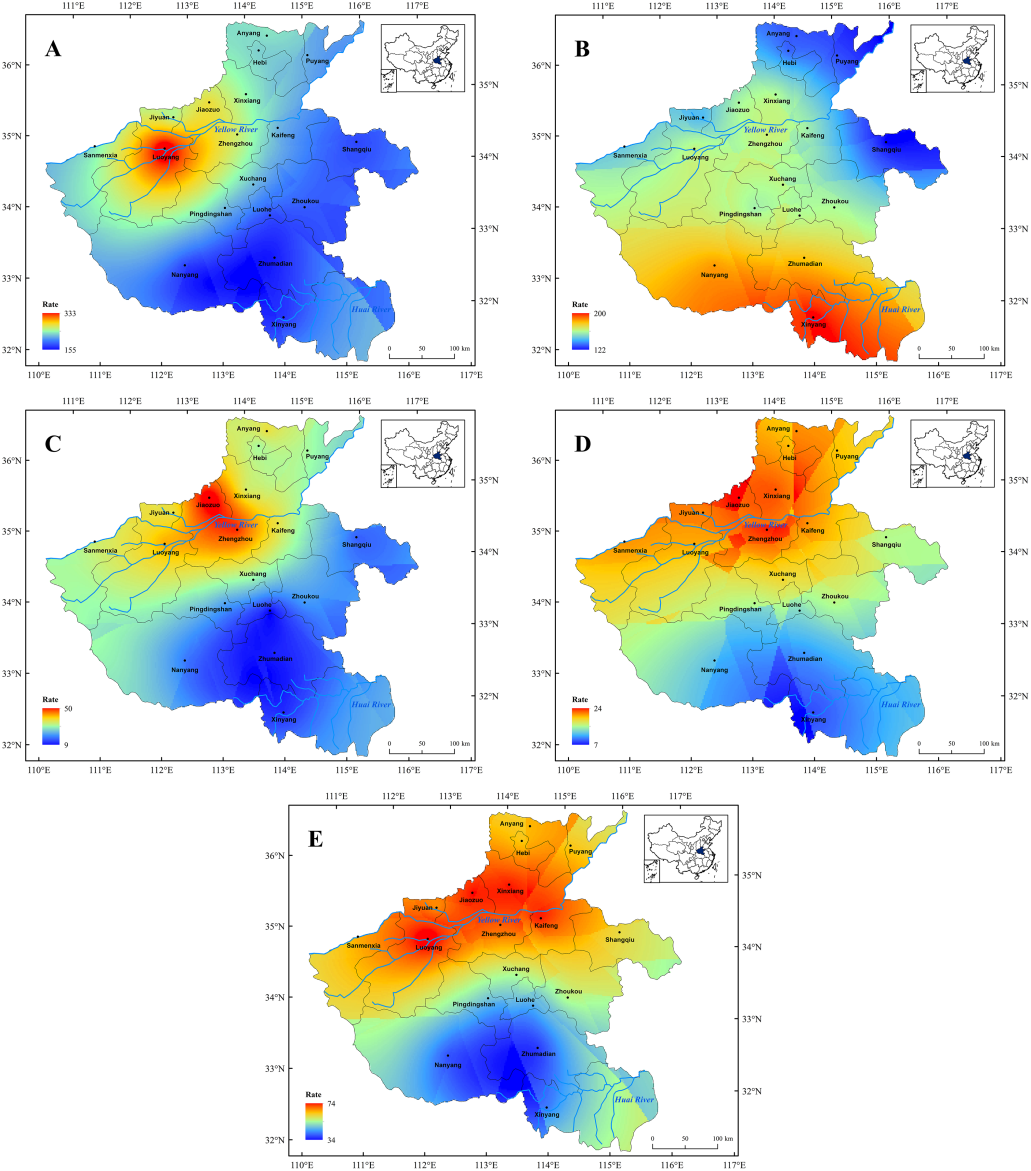
Spatial distribution characters of the major meteorological disasters in Henan Province before 1911 CE. Spatial distribution characters of the major meteorological disasters in Henan Province before 1911 CE. (A) Drought, (B) flood, (C) hail, (D) low temperature and frost, (E) insect pests.

Furthermore, the Henan Province is one key birthplace of ancient Chinese culture, and is known as “the cradle of Chinese civilization” (Shu & Finlayson, 1993; Li, Li & Zhang, 2020). In this region, the cities of Luoyang, Kaifeng and Anyang were the important ancient and densely populated capitals in Chinese history. Historically, Henan Province had been the main agricultural producing area in China (Zheng, Wen & Fang, 2020; Gao, Li & Cui, 2017), and the large-scale low temperature and frost disaster in spring hampered the crops growth, while our statistics data also indicate that low temperature and frost disasters occurred frequently in historical period (Fig. 3). In the past, the drought and insect pests’ disasters have occurred frequently in Henan (Fig. 3), and major impacts were seen in the central and northern part of this region (Figs. 4 and 5), as these parts of the Henan Province were plain farming region. Human beings were vulnerable to drought, insect pests and other meteorological disasters due to the weak resistance to natural disaster and were mostly dependent on heaven for food (Lee et al., 2017; Fang et al., 2015; Wan et al., 2017b). Furthermore, household registration data, the historical population size in the middle and lower Yellow River showed a progressive population growth during the last 2,000 years (Yang, 1993; Zhang, Zhang & Pei, 2021), which corresponds with the increase of the meteorological disasters (Fig. 3). Previous research has demonstrated that the increasing population density resulted in large-scale land reclamation for agriculture in North China (Zheng, Wen & Fang, 2020), and historical documents also showed that many forests and flood plain were cleared and converted to croplands in Henan Province during the Ming and Qing Dynasties (1368–1911 CE) (Zhu, 1991). Therefore, the change of regional environment and the increase of farmland caused the occurrence of meteorological disasters more frequent (Figs. 2 and 3). Meanwhile, due to its location in the middle and lower of the Yellow River, the anthropogenic deforestation and agricultural expansion could also enhance the soil erosion and increase sedimentation rate in the channel, which can easily make the levee prone to breaching and cause widespread flooding (Li, Li & Zhang, 2020; Li et al., 2021b). Our results also proved the flood disaster occurred frequently over the past 2,000 years and increased significantly with the increase in population (Fig. 3). Additionally, we also found that the major meteorological disasters occurred frequently in Henan area during the past 100-year (Figs. 2 and 3). Analyzing the causes and referring to numerous studies, we found that with the increased anthropogenic activities and the continuous development in agricultural and industrial sectors during the last century has greatly accelerated the process of global change and led to the frequent occurrence of extreme meteorological disasters.

In addition to human factors, we compared the occurrence of the meteorological disasters with the climate change. Statistical analysis shows that the occurrence frequency of the major meteorological disasters in Henan Province was roughly the same as the general climate change in eastern China and global climate change during the past two millennia (Figs. 2–4). Previous studies have showed that the duration and amplitude of regional centennial phases of warm/cold fluctuation over the whole China, *i.e.*, warm in 100 BCE–200 CE, 950–1300 CE, 1912–2000 CE and cold in the others, are consistent with that observed in Northern Hemisphere approximately (Figs. 3 and 4). Figures 3 and 4 show the corresponding relationship between warm/cold periods and occurrence frequency of the major meteorological disasters in Henan Province. On the whole, the major meteorological disasters occurred more frequently in cold periods and less in warm periods (Fig. 4). When the climate was in a cold phase, such as the Dark Age Cold period and LIA, meteorological disasters occurred frequently, especially during the LIA (Fig. 4). Based on the historical information, we found that the frequency of the major meteorological disasters was high in the Southern and Northern Dynasties, Five Dynasties, and Ming and Qing Dynasties, when the climate was relatively cold (Fig. 4B). On the contrary, during the warm climate, such as the Roman Warm (Roman W) period and Medieval Warm Period (MWP), the frequency of meteorological disasters was relatively low, such as Tang and Song Dynasties (Figs. 3 and 4B). Therefore, meteorological disasters caused by climate change are an important reason for the frequent disasters in this region. The occurrence of meteorological disasters and the configuration of cold and warm are relatively complex, which are manifested on different time scales, such as decadal or centennial scales. This demands in-depth analysis through a variety of proxy data and model simulation results.

### Relationship between the major meteorological disasters and regional climate change

Meteorological disasters are mainly caused by the imbalance of material and energy in the atmosphere, which is often caused by the background of climate change (Li, Li & Zhang, 2020; Wan et al., 2017c; Zhu, 1991). We compared our record with the reconstructed temperature and humidity records in east China and nearby regions during past two millennia (Fig. 4), which may help understand the occurrence mechanisms of the meteorological disasters in Henan Province on centennial to multi-centennial time scales and help humans mitigate and adapt to natural disaster in the future.

Many scholars have studied the climate change in China over the past 2,000 years and found that the climate change has a great influence on the occurrence of the meteorological disasters in the millennia scale (Wu et al., 2018a; Lee et al., 2017; Zheng, Wen & Fang, 2020; Li, Li & Zhang, 2020; Ge et al., 2016; Gao, Yin & Li, 2019; Hao et al., 2017; Li et al., 2021b). To better understand the impact of climate changes on meteorological disaster in the Henan Province, the major meteorological disaster record was compared with the reconstructed temperature anomaly in east China and North Hemisphere temperatures (Figs. 4A and 4D), and with the dry/wet index in the middle and lower Yellow River basin and nearby regions (Fig. 4C). Previous studies have shown that the temperature changes in the Henan Province were highly consistent with those in most regions of China, whether it is the warming trend in the 20th century or the century-scale warming and cooling phases in the past 2,000 years (Ge et al., 2016; Zheng et al., 2014a; Li et al., 2021b). Among them: during the Han Dynasty (100 BCE–200 CE), Sun and Tang Dynasties (550–850 CE), Song and Yuan Dynasties (950–1300 CE) and the 20th century, the climate was relatively warm and other periods were relatively cold (Figs. 4A and 4B). Therefore, comparing our meteorological disaster records with the reconstructed temperature records and dry/wet index, it reveals that the occurrence of major meteorological disasters in Henan Province were well responded to the climatic changes during past two millennia (Fig. 4).

Our record showed that the frequency of the major meteorological disaster was relatively low prior to 950 CE (Fig. 4B). Although the occurrence of the meteorological disasters was less in this period, it demonstrates variations on centennial time scales (Figs. 3 and 4B) and the timescale of this variability may be determined by patterns of climate (Li et al., 2021b). Before 950 CE, Northern Hemisphere temperature mainly experienced two periods: the Roman Warm Period and the Dark Ages Cold Period, but the temperature phases in east China showed opposite trends with northern hemisphere (Figs. 4A and 4D) (Ge et al., 2012; Ge et al., 2016; Zheng et al., 2014a; Zheng et al., 2021). Our record showed a relatively high frequency of the major meteorological disasters during the cold period from the 50s BCE to the 600s CE (Figs. 4A and 4B), and the high-frequency meteorological disasters in Henan area were coincident with the dry climate on multi-centennial scales based on the dry/wet index in the middle and lower Yellow River basin (Figs. 4B and 4C). Due to the cold and dry climate, we also found that the meteorological disasters were mainly drought and insect pest disaster in this period (Fig. 3). However, a period of high occurrence of the flood disaster could be found in the 280s CE, which was consistent with the wet and warm climate during this period based on the dry/wet index (Fig. 4). Furthermore, during the warm period from the 600s to 950s CE, the occurrences of the major meteorological disasters were relatively stable and had no significant increase (Fig. 4B). This period was marked mainly by flood disaster (Fig. 3). Reconstruction from multiple proxy indicators showed that the climate was warm and humid during this period (Ge et al., 2012; Zheng et al., 2014a), and the Sui and Tang Dynasties were also established in this period (Fig. 4B). Because the climate was suitable and the occurrence of meteorological disasters was less, historical data showed that China’s economy became the first in the world and the number of population increased significantly in this period, which formed the Flourishing Kaiyuan Reign Period (Tian, Stige & Cazelles, 2011). Additionally, we found several periods of significant increases in the occurrence of meteorological disasters before 950s CE, which also occurred during the dynastic transition of China (Fig. 4B), such as the Eastern Han Dynasty (25–220 CE), Three Kingdoms (220–280 CE), Northern and Southern Dynasties (420–589 CE) and the Five Dynasties (907–979 CE). Historical documents showed these dynasties were the relatively long periods of high political and social turmoil in China history (Li et al., 2021a). Based on historical data, the frequency occurrence of the major natural disasters could lead to severe starvation, and then lead to large-scale social unrest and even the change of dynasties (Lee et al., 2017; Fang et al., 2015; Li, Li & Zhang, 2020; Ge et al., 2016). As can be seen from Fig. 4B, the period of dynastic succession in China was also a period of frequent meteorological disasters. Previous studies have shown that the historical famines or peasant uprisings were often related to meteorological disasters on an annual time scale, and severe famines and peasant uprisings were often triggered by severe climatic disasters (Pederson et al., 2014; Buckley et al., 2014; Tian, Stige & Cazelles, 2011; Kelley et al., 2015; He, Su & Fang, 2020).

The frequency of the major meteorological disasters occurrence continues to increase from the 950s to 1400s CE, except for the period of 1150s–1270s CE where no historical records were found (Fig. 4B). Flood disaster was the main meteorological disaster in this period (Fig. 3), and the higher occurrence of floods disaster coincided with the warm and wet climate over this period (Fig. 4). Meanwhile, we also found that the higher occurrence of flood disaster period fell largely within the Medieval Warm Period (Figs. 3 and 4D). Furthermore, previous studies have compared the flooding in middle and lower Yellow River basin with the East Asian summer monsoon and solar activities. Research found that the precipitation variability in the middle and lower Yellow River basin was mainly controlled by the East Asian summer monsoon (Wang & Su, 2013; Morrill, Overpeck & Cole, 2003). Oxygen isotope (δ^18^O) records of stalagmites at Dongge Cave indicated that the East Asian summer monsoon continued to strengthen during this period (Dykosi et al., 2005), and the intensified East Asian summer monsoon would transport more water vapor into the Huai River basin and the middle and lower Yellow River basin, which could increase heavy precipitation and river runoff, and then led to the frequency of floods disaster (Xu & Li, 2019; Hirabayashi et al., 2013).

In contrast to the low frequency of the meteorological disasters occurrence prior to 950 CE, our records show a significantly increasing frequency of the major meteorological disasters occurrence in the Henan Province under the cold and dry climate since 1400s CE (Fig. 4). Numerous studies indicated that the last 600 years was an important period that linked the last millennium together with the recent warming climate (Zhou et al., 2021). And it generally can be divided into two periods, the Little Ice Age (LIA; 1400–1910 CE) and the 20th Century Warming Period (20C WP; 1910–2000 CE) (Fig. 4D). Paleoclimate proxies, climate simulations and historical documents indicated the global climate was in the LIA stage from 1400s CE to the beginning of the 20th century (Ge et al., 2012; Lamb, 1997; Gong & Hameed, 1991), which were prominent cold periods (Fig. 4D) and the climate in China was also relatively cold and dry (Figs. 4A and 4C). The LIA is also known as the Little Ice Age of Ming and Qing Dynasties in China (Fig. 4B). During this period, the climate gradually became dry and cold in eastern China, the precipitation gradually decreased (Ge et al., 2012; Zheng et al., 2014a; Zheng et al., 2021), and the climate showed an unstable change trend (Figs. 4A and 4C). Previous studies have shown that the meteorological disasters occurred frequently during the Ming and Qing Dynasties (Wu et al., 2018b; Ge et al., 2012; Zheng et al., 2014b; Zhou et al., 2021). During the Ming Dynasty, the climate changed from wet to dry in fluctuations in east China as demonstrated in many proxy records as well as historical documents (Figs. 4A and 4C), and at the end of Ming Dynasty, the most serious continuous drought disaster occurred since the Qin and Han Dynasties (Fig. 3). During the Qing Dynasty, the climate was generally humid, but the decadal fluctuations were still very obvious (Figs. 4A and 4C). However, during the climatic alternation period, the occurrence of the meteorological disasters increased significantly, and the major meteorological disaster records also demonstrate an abnormal increasing trend during the warm/cold alternation period in Henan Province (Fig. 4B). After the end of the LIA, a warming trend occurred that lasted into the 20C WP, and within the 20C WP, the globally averaged surface temperatures over land and ocean showed a linear warming trend of 0.85 °C from 1880 to 2012 CE (Intergovernmental Panel on Climate Change (IPCC), 2014; Zhou et al., 2021). According to previous studies, the increased frequency and severity of meteorological disasters can be found during the 20C WP (Shi, Cui & Tian, 2020; Li et al., 2021b; Li, Li & Zhang, 2020; Xing et al., 2020; Zhou et al., 2021), and our record also indicated the major meteorological disasters occurred frequently since 1911 CE (Figs. 3 and 4B). Analyzing the causes, we found that with the intensification of human activities and the continuous development of agriculture and industry since the 20th century, which has greatly accelerated the process of global change and led to the frequent occurrence of extreme meteorological disasters of local areas.

### Spatial variations of the major meteorological disaster in different periods

According to the historical documents, we present a 2,221-year meteorological disaster frequency records in Henan Province from 221 BCE (the beginning of Imperial China) to 2000 CE. After the end of the Qing Dynasty (1911 CE), the widespread emergence of newspapers and other media progressed to disaster recording data more accurate and complete. Therefore, taking 1911 CE (when Republic of China was founded) as the boundary, we divide the frequency of the major meteorological disaster over the past two millennia into two periods (Table 2), and then we analyze the spatial distribution characters and transferred trend of the major meteorological disasters in this region.

### Spatial variations of the major meteorological disaster before 1911 CE

The spatial distribution of major meteorological disasters in Henan Province before 1911 CE is shown in Fig. 5. We found that the meteorological disasters were widely distributed in this region, with a total of 1,598 times, and the frequency of each meteorological disaster indicates significant regional disparities.

(1) Drought disasters showed a decreasing trend from northwest to southeast before 1911 CE in Henan (Fig. 5A). Among them, the drought disaster occurred most frequently in Luoyang City, with a total of 333 times. This is likely due to the geographical location of Luoyang and its status as the ancient capital of the thirteen Dynasties in the history China (Shu & Finlayson, 1993; Li, Li & Zhang, 2020). Luoyang is located in the northwest of Henan Province, which belong to a semi-arid and semi humid area with relatively poor water resources. Due to the monsoon climate, the precipitation showed a seasonal imbalance in yearly distribution and regional discrepancy in spatial distribution, which made the frequent of drought disaster (Zhang et al., 2008). Meanwhile, as the ancient capital of the thirteen Dynasties, Luoyang has a dense population and large agricultural water consumption, which also could reduce the surface runoff and thus resulted to the drought-prone disasters (Xu, Yin & Wang, 2021). Furthermore, we found that the result of drought disaster in Henan Province is consistent with the previous study that using modern instrumental data (Li et al., 2015a; Li, Zhang & Yao, 2015), which also indirectly confirms the accuracy and rationality of the historical data.

(2) Flood disasters showed the characteristics of a large number of times and a wide range of distribution, and that also showed a decreasing trend from south to north before 1911 CE (Fig. 5B). Among them, flood disaster was mainly concentrated in the south of Henan Province, especially in Xinyang City, with a total of 230 times. This is because the upper reaches of Huai River flows through the south of Henan area, and the Huai River basin has always been a frequent area of the flood disaster (Gao, Yin & Li, 2019; Wang, Pan & Chen, 2017; Zhang & Chen, 2020). As influence of the East Asian monsoon, the precipitation is relatively concentrated in this area; meanwhile, there are many tributaries in the Huai River basin, which makes the catchment area very wide. However, due to lack natural waterways into the sea in the lower reaches of the Huai River, that makes it impossible to discharge the flood in time and is easy to form a large-scale water-logging area (Gao, Yin & Li, 2019; Wang, Pan & Chen, 2017; Yang et al., 2014; Sheng et al., 2019).

(3) The hail disaster mainly occurred in the northwest of Henan Province before 1911 CE (Fig. 5C). Occurrence was seen most frequently in Jiaozuo City, with a total of 45 times, followed by Xinxiang and Jiyuan City, while the least number occurred in Luohe City. In the historical period, the northwest of Henan area was densely populated and economically developed. Due to the different nature of disaster bearing bodies in different regions, hail disasters had different damage degrees to different regions as a strong convective weather process (Zhao et al., 2015). The areas with dense population, developed social economy and concentrated human activities were usually the areas with more severe hail disasters (Wan et al., 2017b). In addition, our result showed that the hail disaster has a significant regional feature in Henan Province, especially in mountainous areas (Fig. 5C). A previous study has indicated that the strong atmospheric convection in summer and autumn accelerated the occurrence of hail disasters in mountainous terrain area (Wan et al., 2017a). Therefore, the influence of mountain topography on airflow also made the mountainous areas becoming hails disaster prone area in Henan.

(4) According to statistics, the paper presents that the feature of the low temperature and frost disaster appeared on a diminishing scale from north to the south in Henan area before 1911 CE (Fig. 5D). Among them, Anyang, Luoyang and Xinxiang City were the disaster-prone area, which was almost located in the central and northern parts of the Henan Province (Fig. 1). This is because the central and northern parts of the Henan Province are plain farming region, with a large population and developed agriculture (Zheng, Wen & Fang, 2020; Yang, 1993). However, due to the weak resistance to natural disaster, human beings were vulnerable to meteorological disaster (Lee et al., 2017; Fang et al., 2015; Wan et al., 2017b; Zhang et al., 2014). Therefore, it was extremely vulnerable to low temperature and frost and other meteorological disasters, and the large-scale low temperature and frost disaster in spring has seriously harmed to crops growth, which made the frequency much than that in southern mountainous areas (Zhang et al., 2018a; Zhang et al., 2015).

(5) The occurrence frequency of insect pests had obvious spatial differences in Henan Province before 1911 CE, and presented the spatial distribution characteristics of more in the north and less in the south (Fig. 5E). The insect pests occurred most frequently in Luoyang City, with a total of 72 times, followed by Jiaozuo and Xinxiang City. The result of this study is consistent to the previous study, which also proved that northern part of Henan Province is an area with a high incidence of insect pest (Li et al., 2015c). This character also suggests that central and northern parts of the Henan area, as the major agricultural production area, was extremely vulnerable to meteorological disasters, such as drought, low temperature and frost and insect pests. In addition, previous studies indicated that insect pest and drought disaster displayed a positive correlation, inferring drought would trigger insect pest (Zhang et al., 2012; Li et al., 2015c; Xiao, 2018). Therefore, as we can see in Figs. 5A and 5E, the insect pest is also mainly concentrated in areas with frequent drought disaster, and the occurrence time of the insect pests was consistent with that of drought disasters during the past two millennia.

### Spatial variations of the major meteorological disaster since 1911 CE

**Figure 6:**
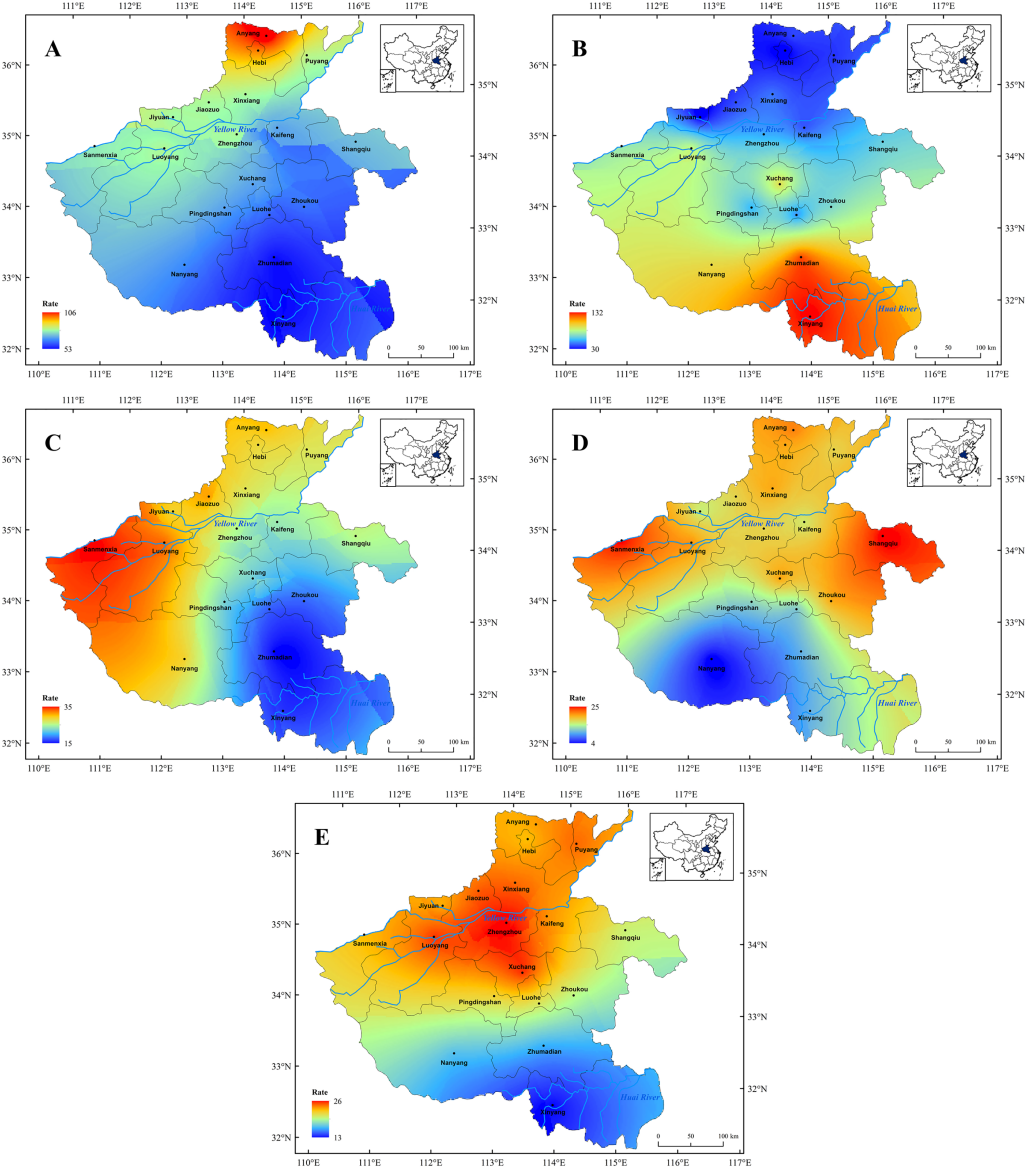
Spatial distribution characters of the major meteorological disasters in Henan Province since 1911 CE. Spatial distribution characters of the major meteorological disasters in Henan Province since 1911 CE. (A) Drought, (B) flood, (C) hail, (D) low temperature and frost, (E) insect pests.

According to the statistics, the major meteorological disasters were recorded 331 times in total since 1911 CE in Henan Province (Table 2) and the spatial distribution of major meteorological disasters is as shown in Fig. 6. As we can see in Table 2, different disasters had significantly different frequencies, flood and drought disaster were still the dominant natural disasters in Henan Province.

(1) Since 1911 CE, the occurrence of drought has changed compared with that before 1911 CE, and the high incidence area was mainly concentrated in the northern part of the Henan Province. Among them, the drought disaster occurred most frequently in Anyang City (Fig. 6A). However, on the whole, it still showed a decreasing trend from northern to southern in Henan. Furthermore, statistics found that drought disaster had occurred most frequently in Henan since 1911 CE. This might be mainly due to the rapid growth of population and the continuous improvement of industrial and agricultural development in Henan Province over past 100 years, which led to the interception of a large amount of surface runoff and made drought disasters frequent (Ma et al., 2019; Chen et al., 2017). Meanwhile, our result is also consistent with the previous studies that employed modern instrumental data in Henan Province (Shi et al., 2015), which also indirectly confirms the accuracy and rationality of the historical documents. In addition, previous studies have shown that climate change has a significant impact on the occurrence of drought disaster (Kundzewica et al., 2014; Zhang et al., 2018b; Van Aslst, 2006). Since 1950s, the temperature of Chinese mainland has gradually increased and entered a modern warming period, especially since 1980s, and the intensification of the warming has promoted the occurrence of extreme drought disasters events (Han, Hao & Zheng, 2013; Wang et al., 2020; Zhang et al., 2021a). Statistics also revealed that drought disasters occurred frequently in Henan Province in the past 50 years (Fig. 3). However, in recent decades, the construction of water conservancy projects has alleviated the occurrence of drought disasters in Henan Province to a certain extent.

(2) Since 1911 CE, the spatial distribution of flood disasters was consistent prior to 1911 CE. The flood disaster was mainly distributed in the south part of Henan Province since 1911 CE, especially the Huai River basin (Fig. 6B), and the flood disaster occurred most frequently in Xinyang and Zhumadian City. It is well-know that the flood disaster is greatly affected by climate, and the topography and geomorphology also have a significant impact (Kundzewica et al., 2014; Lehner et al., 2006; Hirabayashi et al., 2013). The Huai River basin (Henan section) lies in the south of Henan Province, where is also located in the subtropical monsoon climate zone prevails. The precipitation is more concentrated under the influence of the subtropical high belt and the East Asian monsoon (Wang, Pan & Chen, 2017;; Sheng et al., 2019). Furthermore, most areas of the Huai River basin is the gently rolling topography with a height difference of less than 200 m, which does not drain smoothly and is prone to water-logging (Yang et al., 2014). In addition, this result is also in agreement with the previous study of the spatial distribution of historical flood events (Gao, Yin & Li, 2019), which indicate that the Huai River basin has always been plagued by frequent flood disasters, and the flood control measures need to be further strengthened in this region. However, compared with the historical period, the frequency of flood disasters has decreased significantly in Henan Province since 1911 CE, which was directly associated with dike construction and river course dredging of the Yellow River and Huai River in modern times (Yin et al., 2018).

(3) Since 1911 CE, hail disasters occurred less frequently in Henan Province, but the spatial distribution of hail disasters has been of no great fluctuation. Hail disasters occurrence still exhibits strong local characteristics and the high occurrence area of hail disasters is still concentrated in the western mountainous area (Fig. 6C). Meanwhile, this result is consistent with the previous study that employed modern instrumental data in Henan (Yang & Ding, 2015). This might be due to the mountainous terrain and high altitude in the western part of Henan area, which was prone to a short-time strong convective weather in summer, and thus conducive to the occurrence of hail disaster (Zhao et al., 2015).

(4) According to statistical, the scope of the low temperature and frost disaster is relatively scattered in Henan Province since 1911 CE, which still presents the spatial distribution characteristics of more in the north and less in the south, especially in the northern plains (Fig. 6D). Furthermore, our result is consistent with a previous study too, which indicated that the distributions of low temperature and frost disaster were mainly in the plain region, such as in Guanzhong plain (Wan et al., 2017c). Similarly, the northern part of Henan Province is mainly plain terrain, which makes it easier for the Siberian cold snap to penetrate and spread, and then led to the frequent occurrence of low temperature and frost disaster (Gao, Li & Cui, 2017; Shen et al., 2017). The southern and western parts of Henan Province are mainly mountain terrain, such as Tai-hang Mountain, Fu-niu Mountain and Ta-pieh Mountains (Fig. 1). The higher mountain act as a barrier in blocking the Siberian cold snap, and thus the temperature fluctuation is relatively small (Gao, Li & Cui, 2017). Therefore, the occurrence frequency of low temperature and frost disaster event is rare. In addition, due to the intense human activities, global extreme weather events occurred frequently since the 1950s (Du et al., 2013), which led to more frequent frost disasters (Fig. 3B). Therefore, in order to reduce the impact of frost disasters, people need to constantly adjust the regional agricultural industrial structure in the future.

(5) Since 1911 CE, the high incidence area of insect pests was similar to that before 1911 CE, which mainly occurred in the plain farming areas in the central and northern of Henan Province, while were less frequent in the southern mountainous areas (Fig. 6E). Furthermore, compared with the historical period, we found the occurrence of the insect pests decreased significantly since 1911 CE, which was mainly due to the continuous development of modern disaster prevention and mitigation technology (Leng & Hall, 2019). Meanwhile, the plant protection policy of “prevention first and prevention combined” is conducive to strengthening the monitoring of the insect pests diseases (Liu, Yang & Li, 2012), which has effectively controlled the occurrence of the insect pests diseases in Henan Province in the past 100-years. In addition, previous studies indicated that insect pests could obviously correspond to the long-term climate change; the climate-transition period and the partial dry warm period were the main causes of the large-scale insect pests (Li et al., 2015b; Xiao, 2018). This characteristic has been well reflected in Henan Province, that the insect pest disaster has a strong relationship with precipitation and presents a positive correlation with drought (Fig. 6).

### Comparison of the spatial pattern of the major meteorological disaster before and after 1911CE

Taking 1911 CE as the boundary, we analyze the correlation between the occurrence frequency of the major meteorological disaster before 1911 CE and that of since 1911 CE. Finally, we obtained the spatial distribution characters and transferred trend of each major meteorological disasters in a long-time scale in the Henan area (Table 3). The correlation analysis results of spatial distribution changes of meteorological disasters has been presented in the Table 3 where the significant correlation showed that there is no obvious transfer in the disaster-prone area, while the non-correlation indicates the obvious transfer in the disaster-prone area.

**Table 3:** Correlation coefficient between the number of various meteorological disasters before and after 1911 CE in the same region.

Types of disaster	Before 1911 CE and Since 1911 CE			
	Significance (99% confidence interval)	Sig*	Significance (95% confidence interval)	Sig**
Drought	×	0.394	×	0.13
Flood	√	0.05	√	0.014
Hail	×	0.141	√	0.017
Low temperature and frost	×	0.18	√	0.004
Insect pests	√	0.035	√	0.002

**Note:**

√Significant correlation; × no correlation. *p < 0.05, **p < 0.01.

The results of correlation analysis of the each meteorological disaster in different regions before 1911 CE and during 1911–2000 CE in Henan area showed that:

(1) The correlation coefficient of drought disaster was 0.394, which indicated that the main occurrence area of drought disaster has transferred in these two periods (Table 3). Before 1911 CE, the frequency-occurring district of drought disaster was mainly concentrated in the northwest of the Henan area, among which Luoyang and Jiyuan City were greatly influenced by drought disasters (Fig. 5A). After that, the high incidence area of the drought disaster has transferred to the northward of the Henan area, which mainly concentrated in Anyang and other places in the north of the Henan area. (2) The correlation coefficient of the hail, low temperature and frost disaster were 0.536 and 0.567 in these two periods, respectively, which was significantly correlated at 95% confidence interval, but not at 99% confidence interval (Table 3). The result showed that the high incidence areas of hail, low temperature and frost disaster occurred a little shift in these two periods, but the change was not obvious. (3) The correlation coefficient of flood and insect pest disaster were 0.597 and 0.617 in these two periods, respectively, which was significantly correlated both at 95% and 99% confidence interval (Table 3). This result indicates that the spatial distributions of these two disasters are consistent since the historical records, and there is no obvious shift in the disaster prone areas.

## Conclusions

We present a long-term documentary record of the major meteorological disasters events in Henan Province during the last 2,000 years, in an attempt to better understand of the spatio-temporal pattern of the meteorological disasters in this region and the underlying climatic and anthropogenic drivers. A summary of the main findings are follows:

(1) Meteorological disasters have occurred frequently in Henan Province during the past 2,000 years. Among them, drought and flood disaster occurred most frequently. Statistics revealed that the frequency of the major meteorological disasters exhibits centennial-scale variability in Henan area. And the anthropogenic began to play a dominant role in the significant increase of the extreme meteorological disasters frequent since the 20^th^ century.

(2) The occurrence of major meteorological disasters have well corresponded to the climate change in Henan Province during the past two millennia. That is, the occurrence of major meteorological disasters frequency was high in the cold period, and was low in the warm period. Furthermore, the major meteorological disaster records also demonstrated an abnormal increasing trend during the warm/cold alternation period.

(3) The occurrence of major meteorological disasters presents obvious spatial differences in Henan Province during the past two millennia. The northern and western parts of the Yellow River Basin were prone to drought disaster. On the contrary, the flood disasters were mainly distributed in the southern part of the Henan area, especially in the Huai River Basin, while the low temperature and insect pest disasters mainly distributed in the central and northern of this region.

(4) Due to the increased anthropogenic activities and global change, the high incidence area of the drought disaster has transferred to the northward of the Henan Province after 1911 CE, while the frequency-occurring district of flood and insect pest disaster have not been obviously transferred. Meanwhile, the high incidence areas of hail, low temperature and frost disasters just have a smaller transferred after 1911 CE.

## Supplemental Information

10.7717/peerj.12365/supp-1 Supplemental Information 1 Collection of meteorological disasters in Hena area during the past two millennia.

## Additional Information and Declarations

### Data Availability

The following information was supplied regarding data availability: The raw measurements are available in the Supplementary Files.
